# Metabolic Changes Induced by Deletion of Transcriptional Regulator *GCR2* in Xylose-Fermenting *Saccharomyces cerevisiae*

**DOI:** 10.3390/microorganisms8101499

**Published:** 2020-09-29

**Authors:** Minhye Shin, Soo Rin Kim

**Affiliations:** 1 Department of Agricultural Biotechnology, Research Institute of Agriculture and Life Science, Seoul National University, Seoul 08826, Korea; mhshin1984@snu.ac.kr; 2 School of Food Science and Biotechnology, Kyungpook National University, Daegu 41566, Korea

**Keywords:** *GCR2*, glycolysis, pentose phosphate pathway, xylose, metabolomics

## Abstract

Glucose repression has been extensively studied in *Saccharomyces cerevisiae*, including the regulatory systems responsible for efficient catabolism of glucose, the preferred carbon source. However, how these regulatory systems would alter central metabolism if new foreign pathways are introduced is unknown, and the regulatory networks between glycolysis and the pentose phosphate pathway, the two major pathways in central carbon metabolism, have not been systematically investigated. Here we disrupted *gcr2*, a key transcriptional regulator, in *S. cerevisiae* strain SR7 engineered to heterologously express the xylose-assimilating pathway, activating genes involved in glycolysis, and evaluated the global metabolic changes. *gcr2* deletion reduced cellular growth in glucose but significantly increased growth when xylose was the sole carbon source. Global metabolite profiling revealed differential regulation of yeast metabolism in SR7-*gcr2Δ*, especially carbohydrate and nucleotide metabolism, depending on the carbon source. In glucose, the SR7-*gcr2Δ* mutant showed overall decreased abundance of metabolites, such as pyruvate and sedoheptulose-7-phosphate, associated with central carbon metabolism including glycolysis and the pentose phosphate pathway. However, SR7-*gcr2Δ* showed an increase in metabolites abundance (ribulose-5-phosphate, sedoheptulose-7-phosphate, and erythrose-4-phosphate) notably from the pentose phosphate pathway, as well as alteration in global metabolism when compared to SR7. These results provide insights into how the regulatory system *GCR2* coordinates the transcription of glycolytic genes and associated metabolic pathways.

## 1. Introduction

Glucose repression, a phenomenon whereby cells grown on glucose repress the metabolism of alternate carbon sources, has been extensively studied and established in *Saccharomyces cerevisiae* [1]. The glucose repression pathway is a complex network of signals and regulations where native regulator systems adjust the yeast cellular metabolism and fermentation profiles depending on the available carbon source. Understanding the mechanism and regulation of glucose metabolism in yeast is critical for the development of biotechnological processes to redirect the carbon fluxes toward products of interest [2].

The *S. cerevisiae* Gcr2 is a transcriptional activator that regulates expression of most glycolytic genes [3]. Gcr2 protein enhances the CT box-dependent transcriptional activation of a RAP1-GCR1 complex required for the expression of glycolytic genes [4]. As a transcriptional activation complex, RAP1-GCR1 provides the specific DNA-binding and the activation of glycolytic and ribosomal genes [5,6]. Rap1 is a multifunctional DNA-binding protein that activates transcription of glycolysis genes and translational components. Rap1 acts interdependently with Gcr1, which mediates transcription of its target genes, along with Rap1 by providing an activation domain [7]. Gcr2, the other component of the activation complex, provides an axillary activation domain to the GCR1-GCR2 complex, mediating high level of glycolytic gene expression [3]. However, the mechanism by which the regulatory systems function in the presence of an alternative carbon source and the working of the system in the case of introduction of new foreign pathways, such as the heterologous xylose-assimilating genes, is currently unknown. Finally, the regulatory networks between glycolysis and the pentose phosphate pathway, the two major pathways in central carbon metabolism, have not been systematically investigated.

A global system-based approach to investigate changes in small-molecule metabolite profiles is an important tool to understand the phenotypes and fermentation outcomes in biological processes. Recently, high-resolution mass spectrometry has been used to elucidate the metabolic interactions, enabling a better understanding of the biological systems and regulations utilized under different physiological conditions [8]. In addition, the development of analytical and associated statistical methods allow high-throughput screening of hundreds of metabolites from complex biological samples, providing integrative information of the functional status of the cell in response to either genetic or environmental changes [9].

Here, we systemically investigated the regulatory mechanism of *GCR2* in the *S. cerevisiae* SR7-*gcr2Δ* strain engineered for xylose metabolism at the metabolomic level. We provide extensive evidence for how global metabolic pathways are affected in the SR7-*gcr2Δ* strain when grown on different carbon sources, namely glucose and xylose. We found that the genetic alteration caused differential regulation of yeast metabolism, depending on the available carbon source, and the metabolic changes ultimately resulted in growth improvement via the activated pentose phosphate pathway when cells were grown on xylose as a sole carbon source. This finding suggests that native regulator systems, primarily transcriptional regulations, are highly associated with suboptimal xylose fermentation by xylose-fermenting *S. cerevisiae*, improving a highly efficient biotechnological process toward the generation of products of interest.

## 2. Materials and Methods

### 2.1. Strain Construction

We constructed a xylose-fermenting *S. cerevisiae* D452-2 strain (SR7) using the linear expression cassette of *Scheffersomyces stipitis XYL1*, *XYL2*, and *XYL3* genes as described previously (Table 1) [10]. To construct the *gcr2* mutant, the *gcr2*::KanMX4 cassette was amplified from the genomic DNA of the BY4742 *gcr2* strain (clone ID: 12013) of the Yeast Knockout Collection (Thermo Fisher Scientific, Waltham, MA, USA) by polymerase chain reaction (PCR) using *GCR2*-specific primers (upstream: 5′-CAACCCTATGCTACAAGAGCAG-3′ and downstream: 5′-CGACACTAAACCCAGCTAACTC-3′). The PCR product was purified and genome-integrated to the SR7 strain by the LiAc transformation method [11]. The resulting deletion mutant was selected on an agar medium containing 10 g/L yeast extract, 20 g/L peptone, 20 g/L glucose (YPD), 15 g/L agar, and 300 mg/mL G418 sulfate (GoldBio, St. Louis, MO, USA).

### 2.2. Culture Conditions and Fermentation Experiments

Yeast cells were pre-cultured in 5 mL of YPD medium (10 g/L yeast extract, 20 g/L peptone, and 20 g/L glucose) for 24 h at 30 °C and 250 rpm. Cells were harvested by centrifugation at 15,000 rpm for 1 min. The cells were adjusted to a concentration of OD_600_ 1.0 (OD_600_ 1.0 = 3.5 × 10^7^ cells/mL) and into inoculated 20 mL of YPD (20 g/L glucose) or YPX (20 g/L xylose) in a 96-well microplate at 30 °C. Cell growth was monitored at 600 nm using a spectrophotometer (OD_600_). All experiments were repeated in triplicate.

### 2.3. Intracellular Metabolite Extraction

Metabolite extraction was conducted according to the method described previously [13]. Briefly, a colony of yeast cells was pre-cultured in 5 mL YPD for 24 h at 30 °C and 250 rpm. Cells were harvested by centrifugation at 15,000 rpm for 1 min. The cells were adjusted to a concentration of OD_600_ 1.0 (OD_600_ 1.0 = 3.5 × 10^7^ cells/mL) and inoculated in 50 mL YPD (40 g/L glucose) or YPX (40 g/L xylose) in a 250 mL Erlenmeyer flask at 30 °C and 80 rpm. Five milliliters of cell culture at mid-exponential growth phase (8 h and 10 h of incubation for SR7 and SR7-*gcr2Δ* grown on glucose, respectively; 72 h and 48 h of incubation for SR7 and SR7-*gcr2Δ* grown on xylose, respectively) were quenched by quick injection into 25 mL of 60% (*v*/*v*) cold methanol (HEPES, 10 mM; pH 7.1) at −40 °C. The cells were centrifuged at 4000 rpm (3134× *g*) for 10 min at −20 °C and the supernatant was completely removed. Subsequently, 75% (*v*/*v*) boiling ethanol (HEPES, 10 mM; pH 7.1) was added to the quenched cell pellet. The mixture was vortexed for 30 s, incubated for 5 min at 80 °C, and immediately cooled in an ice-bath for 5 min. The cell residues were separated from the extract by centrifugation at 4000 rpm (3134× *g*) for 10 min at 4 °C. The supernatant was vacuum-dried for 6 h using a speed vacuum concentrator.

### 2.4. Derivatization of Metabolites and Metabolite Analysis Using GC/MS

Prior to GC/MS analysis, the vacuum-dried samples were derivatized by methoxyamination and trimethylsilylation. For methoxyamination, 40 µL of methoxyamine hydrochloride in pyridine (40 mg/mL; Sigma-Aldrich, St. Louis, MO, USA) was added to the samples and incubated for 90 min at 30 °C. For trimethylsilylation, 40 µL of *N*-methyl-*N*-trimethylsilyltrifluoroacetamide (Sigma-Aldrich, St. Louis, MO, USA) was added to the samples and incubated for 30 min at 37 °C.

GC/MS analysis was conducted using an Agilent 6890 GC equipped with Leco TOF. A 1 µL aliquot of derivatized samples was injected into the GC in a splitless mode and separated on an RTX-5Sil MS column (30 m × 0.25 mm, 0.25 µm film thickness; Restek, Bellefonte, PA, USA). The initial oven temperature was set at 50 °C for 1 min, and then ramped at 20 °C/min to a final temperature of 330 °C, held for 2 min. Helium was used as a carrier gas at a constant flow rate of 1.5 mL/min. The temperatures of ion source and transfer line were set at 250 °C and 280 °C, respectively. An electron impact of 70 eV was used for ionization. The mass selective detector was operated in scan mode with a mass range of 50–800 *m*/*z*.

### 2.5. Statistical Analysis

The metabolites were evaluated by principal component analysis (PCA) using STATISTICA software (Version 7.0, Stat Soft, Tulsa, OK, USA). The presented data were based on significance (*p* < 0.05) by *t*-test using GraphPad Prism 8 (San Diego, CA, USA). Metabolites were mapped into a biochemical network using the MetaMapp software [14]. Fold changes were mapped to node size, and direction was mapped to node color, where blue and red mean lower and higher metabolites abundance, respectively. Label font size indicates *p*-value by *t*-test. Hierarchical clustering was conducted using ClustVis (version 2.0, https://biit.cs.ut.ee/clustvis), a web tool for visualization of multivariate data clustering based on PCA and heatmap [15].

## 3. Results

### 3.1. Deletion of gcr2 Enhances Growth of the Engineered S. cerevisiae SR7-gcr2Δ on Xylose

**Figure 1:**
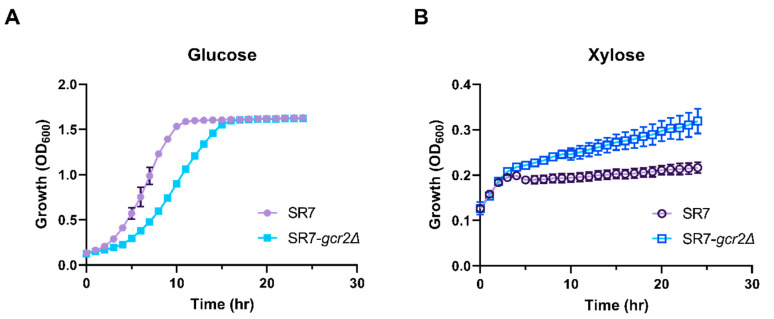
Growth profiles of *S. cerevisiae* SR7 and SR7-*gcr2Δ* on glucose (**A**) and xylose (**B**) media. Cell density was measured by optical density at 600 nm. All of the experiments were performed in triplicate. The error bars represent the standard deviations. ●: *S. cerevisiae* SR7; and ■: *S. cerevisiae* SR7-*gcr2Δ*.

*GCR2* is a transcriptional regulator that upregulates glycolytic gene expression, especially at the energy payoff phase of glycolysis [5]. We hypothesized that SR7-*gcr2Δ* would reduce glycolytic activity, resulting in stimulation of the non-oxidative pentose phosphate pathway, when xylose is used as the alternative carbon source. To test this hypothesis, we characterized the phenotypic change induced by SR7-*gcr2Δ by* comparing it with the wild-type strain of *S. cerevisiae* SR7 grown using either glucose or xylose as the sole carbon source. The SR7 strain is an engineered *S. cerevisiae* D452-2 with heterologous expression of the xylose-assimilating pathway genes from *Scheffersomyces stipitis* XYL1, XYL2, and XYL3, encoding xylose reductase, xylitol dehydrogenase, and xylulokinase, respectively [10,16]. As expected, when the strains were grown on glucose, the SR7-*gcr2Δ* strain showed slower growth than the SR7 strain (Figure 1A, specific growth rate 1.78 ± 0.35 h^−1^ and 0.87 ± 0.03 h^−1^ for SR7 and SR7-*gcr2Δ*, respectively). However, when the strains were grown on xylose, the SR7-*gcr2Δ* mutant grew significantly faster than SR7 strain (Figure 1B, specific growth rate 0.012 ± 0.004 h^−1^ and 0.052 ± 0.001 h^−1^ for SR7 and SR7 SR7-*gcr2Δ*, respectively). These data suggest that *gcr2Δ* triggers transcriptional alteration of glycolysis and the associated pentose phosphate pathway, which directly improves xylose metabolism in the engineered *S. cerevisiae* strain.

### 3.2. Global Metabolic Changes Are Induced in SR7-gcr2Δ

**Figure 2:**
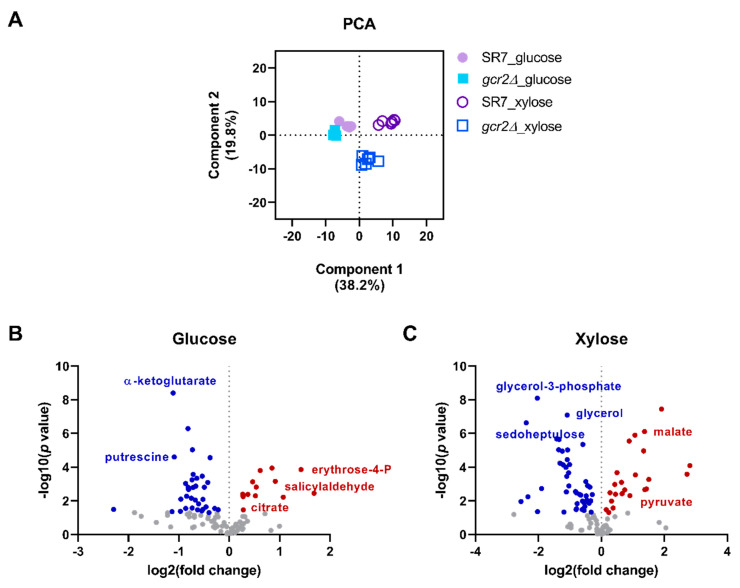
(**A**) Principal component analysis of *S. cerevisiae* metabolites. Intensities of metabolites in SR7 and SR7-*gcr2Δ* grown on glucose and xylose media were detected by GC-TOF MS. (**B**,**C**) Volcano plot analysis of identified metabolites on glucose (**B**) and xylose (**C**). The scatter plot shows features with fold change (x) and *t*-test threshold (y) of 0.05, and both values were log-transformed by 2 and 10, respectively. The red or blue dots indicate significantly increased or decreased metabolites in SR7-*gcr2Δ* compared with SR7 (*p* < 0.05), respectively.

To understand the global metabolic changes in SR7-*gcr2Δ*, untargeted metabolite profiling was performed using GC-TOF MS platform with cultures of SR7 and the SR7-*gcr2Δ* mutant grown on glucose and xylose, respectively. As shown in Supplementary Table S1, a total of 110 intracellular metabolites were identified including amino acids, polyamines, organic acids, sugars, and free fatty acids. The hierarchical clustering and PCA analysis showed that the carbon source in the culture media was the primary determinant of the metabolic changes, and the metabolism of SR7-*gcr2Δ* mutant grown on xylose was different from SR7 (Supplementary Figure S1). The PCA score plot exhibited a good fit of R^2^X, 0.58, and a prediction of Q^2^, 0.69, based on the cumulative values up to principal component 2, showing a separation among the metabolite profiles of the four groups (Figure 2A). Especially, the PCA plot shows that the range of metabolic changes in the SR7-*gcr2Δ* mutant was greater than SR7 when grown on xylose. Separation of the plots in the PCA indicates significant alteration of global metabolism in the strain. The PCA result suggests that Gcr2 would affect the xylose metabolism and in turn the associated downstream metabolic pathways more than other metabolic conditions. The volcano plots comparing the detected metabolites between the two strains grown on each carbon source clearly show significantly different metabolic markers based on the statistical significance of the magnitude of the changes (Figure 2B,C). The metabolomics analysis corroborated the results from the growth analysis, showing that the deletion of *gcr2* in *S. cerevisiae* SR7 background induces global changes in yeast metabolism.

### 3.3. Deletion of gcr2 Differentially Alters Yeast Metabolism Depending on Carbon Sources

**Figure 3:**
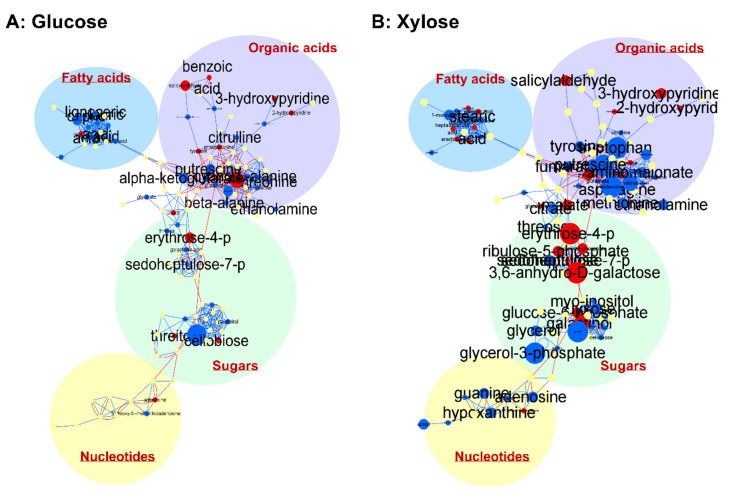
Network analysis of metabolites on glucose (**A**) and xylose (**B**). Each node is a name of structurally identified metabolite. Red edges are KEGG reactant pair annotation, and blue edges are chemical similarity annotations. The graph was visualized using organic layout in Cytoscape. Red nodes are increased metabolites, and blue nodes are decreased metabolites. The size of nodes and labels reflect fold-changes (bigger nodes mean higher fold-changes) and *p* values assigned by *t*-test (bigger font sizes mean more statistically significant changes), respectively. The metabolites were clustered into fatty acids, organic acids including amino acids, sugars, and nucleotides.

In addition to the multi- and univariate analyses, we generated maps to visualize the integrative metabolic network for a better understanding of the SR7-*gcr2Δ* effect with respect to the carbon sources. Using MetaMapp, which helps visualize metabolomics data by integrating information from biochemical pathways and chemical and mass spectral similarity, the metabolites relation network was visualized by CytoScape (Figure 3) [14]. In the depicted network, large sizes of font and circles indicate statistical significance and fold change in the strain SR7-*gcr2Δ*, respectively. On the map, a clear interrelationship between SR7-*gcr2Δ* and metabolic pathways was evident. Specifically, for both glucose and xylose media, the SR7-*gcr2Δ* mutant showed significantly decreased abundances of fatty acids and organic acids, including 1-monopalmitin, salicylaldehyde, and hydroxypyrimidines. However, compared to the glucose metabolism, the SR7-*gcr2Δ* grown in the presence of xylose showed significant changes in metabolite levels. Among the changed metabolites, the abundance of sugars and their derivatives were notably affected by the mutation. Nucleotides levels, such as guanine, hypoxanthine, inosine, and uracil, were also significantly decreased in SR7-*gcr2Δ* grown in xylose, while growth in glucose did not show a significant difference between the two genotypes. Taken together, the depicted network clearly showed that the relationship between SR7-*gcr2Δ* and associated metabolites changes, depending on the available carbon source.

### 3.4. Deletion of gcr2 Alters Pathways in Central Carbon Metabolism

**Figure 4:**
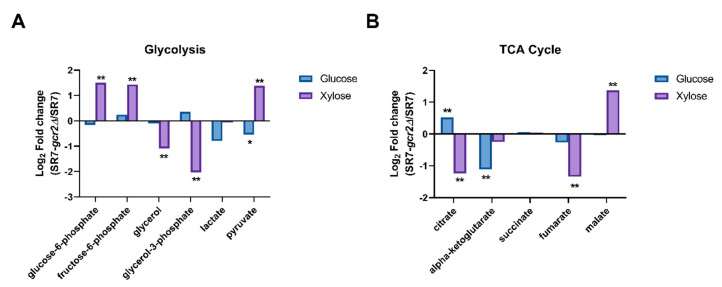
Intracellular metabolite fold-changes in glycolysis (**A**) and TCA cycle (**B**) of SR7 and SR7-*gcr2Δ* grown on glucose (■) or xylose (■). Intracellular metabolites were detected using GC-TOF MS, and fold-changes were converted to a log 2 scale. Statistical analysis was performed using *t*-test, and differences were considered significant when *p* was < 0.01 (**) or 0.05 (*).

Genetic studies have shown that the expression of most glycolytic enzymes is severely reduced in the SR7-*gcr2Δ* mutant and may regulate gene expression in the downstream pathways, such as the TCA cycle and respiration [17]. However, the genetic effect is unknown at the metabolic phenotype level, especially in the presence of an alternative carbon source. With respect to glycolysis, we found that the intracellular abundance of metabolites in the central carbon metabolism was not significantly different between the two strains grown on glucose medium, except for pyruvate (Figure 4). However, levels of glucose-6-phosphate, fructose-6-phosphate, and pyruvate were significantly higher in the presence of xylose. Byproducts of glycolysis, including glycerol and glycerol-3-phosphate, were reduced in the mutant strain grown in xylose. For the TCA cycle, we could detect intermediates in the pathway, such as citrate, alpha-ketoglutarate, succinate, fumarate, and malate (Figure 4B). The abundance of most of these metabolites decreased in SR7-*gcr2Δ*, but citrate and malate intermediates showed particularly higher abundance in glucose and xylose, respectively. Overall, the xylose culture provided a greater impact of SR7-*gcr2Δ* on glycolytic genes than the glucose culture, and central carbon metabolism was differentially affected by the deletion of *gcr2,* depending on the type of sugar available.

### 3.5. Deletion of gcr2 Upregulates Pentose Phosphate Pathway on Xylose

**Figure 5:**
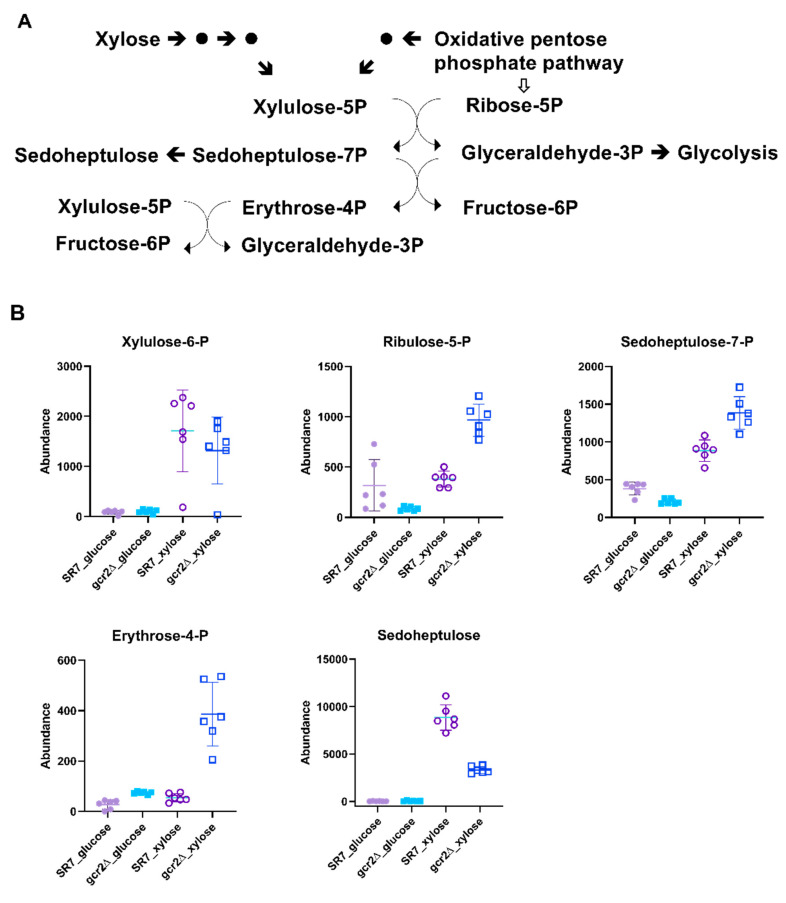
(**A**) The pentose phosphate pathway. (**B**) Abundances of the intermediate metabolites in the pentose phosphate pathway of SR7 (●) and SR7-*gcr2Δ* (■) grown on glucose (closed symbols) or xylose (open symbols). The error bars indicate standard deviation, and all experiments were performed in six replicates.

The pentose phosphate pathway is a universal metabolic pathway producing xylulose-5-phosphate as a key metabolic core intermediate, linking xylose assimilation, an oxidative phase, and a non-oxidative phase, in the pathway [18]. We could detect xylulose-5-phosphate, ribulose-5-phosphate, sedoheptulose-7-phosphate, erythrose-4-phosphate, and sedoheptulose as intermediates in the pentose phosphate pathway. All these metabolites showed higher abundance when cells were grown on xylose than on glucose (Figure 5). For xylose assimilation, there was no significant difference in xylulose-5-phosphate levels between the SR7 and SR7-*gcr2Δ* strains, implicating that the heterologous steps were unaffected in SR7-*gcr2Δ*.

Unlike xylulose-5-phosphate, the metabolic abundances of ribulose-5-phosphate, sedoheptulose-7-phosphate, and sedoheptulose in the oxidative and the non-oxidative phases were differentially regulated in SR7-*gcr2Δ*, depending on the carbon source. Higher levels of these metabolites indicated increased metabolic flow, via the pentose phosphate pathway of the SR7-*gcr2Δ* mutant grown in xylose. Notably, the abundance of erythrose-4-phosphate increased in SR7-*gcr2Δ* grown in both carbon sources, although the absolute abundance was much higher with xylose than with glucose. Overall, this study showed that the deletion of *gcr2* induced the metabolic flow toward the pentose phosphate pathway and improved cellular growth when only xylose was available. In many xylose-fermenting yeast strains, the enhanced flux through the pentose phosphate pathway induces greater xylose utilization and ethanol production [10,16,18]. We speculate that the higher abundance of the intermediate results from downregulation of glycolysis, accumulating erythrose-4-phosphate in SR7-*gcr2Δ*. These results suggest that the glycolytic transcription activator *GCR2* has a negative impact on xylose fermentation, more specifically, on the pentose phosphate pathway, thereby, ultimately providing improved xylose utilization and ethanol production in the SR7-*gcr2Δ* mutant.

### 3.6. Deletion of gcr2 Increases Cellular Defense against Oxidative Stress

**Figure 6:**
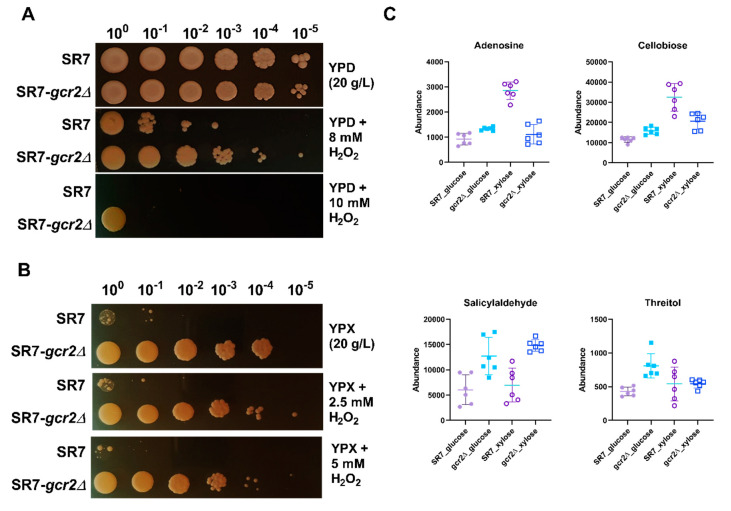
Spot plate assay for SR7 and SR7-*gcr2Δ* grown on glucose (**A**) and xylose (**B**) under control and in the presence of hydrogen peroxide (H_2_O_2_). (**C**) Abundances of the metabolites associated with the cellular antioxidant activity of SR7 (●) and SR7-*gcr2Δ* (■) grown on glucose (closed symbols) or xylose (open symbols). The error bars indicate standard deviation, and all experiments were performed in six replicates.

Inhibition of glycolysis is known to promote flux into the pentose phosphate pathway to generate NADPH. The pentose phosphate pathway is strongly associated with cellular defense against oxidative stress by producing NADPH, which provides the reducing power fueling the antioxidant systems, and recycles oxidized glutathione [19]. We hypothesized that downregulation of glycolysis in SR7-*gcr2Δ* would alter the cellular redox homeostasis during oxidative stress. To test our hypothesis, we pre-cultured the two strains in YPD broth for 24 h and adjusted the optical density of the culture to one. Then, we spotted culture dilutes onto 20 g/L YPD or YPX agar plates, respectively, with supplementation of hydrogen peroxide. As shown in Figure 6, when cells were treated with hydrogen peroxide, the SR7-*gcr2Δ* mutant showed superior cell viability than SR7 on glucose medium. The mutant yeast strain also exhibited improved viability on xylose, however, this effect also could be due to better xylose utilization, not solely from the antioxidant activity.

In addition to the pentose phosphate pathway, we compared the abundance of other metabolites known for their antioxidant activity in yeast, such as adenosine, cellobiose, salicylaldehyde, and threitol [20,21,22,23]. All these metabolite levels were significantly higher in the mutant strain, especially in glucose medium (Figure 6C). In xylose, only the abundance of salicylaldehyde increased in SR7-*gcr2Δ*, while other metabolite levels were unchanged, or even decreased, suggesting the possibility of a different antioxidant mechanism based on the carbon sources. Central carbon metabolism involves a network of interrelated pathways that provide various redox cofactors and generate energy sources. The complicated metabolic network affects different cellular responses as means of the cofactors and energies. Our results showed higher antioxidant activity of SR7-*gcr2Δ* when compared to its wild type, which may be attributed to either the increased pentose phosphate pathway, inhibited glycolysis, or unidentified defense mechanisms against oxidative stress. Taken together, the deletion of *gcr2* induces cells to adjust and reprogram their cellular responses, caused by environmental disruption beyond glycolysis.

## 4. Discussion

Gcr2 is a glycolytic gene transcriptional activator that regulates the expression of most glycolytic genes, in a complex with RAP1 and GCR1. Here, we report that deletion of *gcr2* in xylose-fermenting *S. cerevisiae* improved cellular growth via upregulation of the pentose phosphate pathway. Metabolomic analysis showed global metabolic changes were induced by the deletion of *gcr2*, as well as altered central carbon metabolism. The SR7-*gcr2Δ* strain showed a clearly activated pentose phosphate pathway when grown in the presence of xylose and affected yeast cellular antioxidant activity, especially in glucose, suggesting the global effects of the transcriptional activator beyond glycolysis.

Transcription factors regulate global cellular responses and are, therefore, frequently targeted in the field of synthetic biology and metabolic engineering. To date, a few studies have succeeded to improve ethanol fermentation in yeast by deleting or over-expressing transcription factor genes, such as *HAA1, WHI2, YAP1, NRM1,* and *CAT8* [24,25,26,27,28]. These transcription factors are involved in expression regulation of membrane proteins (Haa1), coordination of growth and stress responses (Whi2), redox homeostasis (Yap1), cell-cycle-dependent transcription (Nrm1), and gluconeogenesis (Cat8), all resulting in higher growth in xylose and enhanced ethanol yield and productivity. In the same context, when xylose was provided as a carbon source, the deletion of *gcr2* effectively shifted the metabolic flux toward the pentose phosphate pathway, resulting in improved xylose utilization and cellular growth. Since *GCR2* regulates glycolysis, the central cellular carbon metabolism was greatly affected. Yeast metabolic pathway will be mainly directed to the pentose phosphate pathway from xylose in the xylose culture. The intermediates in the pathway are fructose-6-phosphate and glyceraldehyde-3-phosphate shared by glycolysis, which are key components especially for the second phase of glycolysis. Therefore, we speculate that deletion of *gcr2* would greatly impact the pentose phosphate pathway in SR7-*gcr2Δ*, resulting in subsequent changes in glycolysis and overall central carbon metabolism. Notably, we speculate that the changes in the metabolic profiles shown in the current study may have resulted from using xylose as an alternative carbon source and not from the absence of glucose in the medium. Sasaki et al. reported that the deletion of *gcr2* in *S. cerevisiae* did not affect the cellular growth nor the expression of genes in TCA cycle and oxidative phosphorylation when cells were grown on glycerol plus lactate as alternative carbon sources of glucose [17]. Therefore, the metabolic outcomes present here would be the effect of xylose as an alternative carbon source. Additional transcript expression profiles in these pathways need to be investigated in SR7-*gcr2Δ* to further understand the role of *GCR2* in SR7.

Although there have been a number of successful strategies to implement xylose metabolism in *S. cerevisiae*, their efficiency so far has not been comparable to that of glucose [10,18]. Xylose paradox is a paradoxical phenomenon in the engineered yeast that do not sense xylose as a fermentable carbon source despite being able to grow on and ferment xylose [29]. Many studies showed that high xylose concentrations would trigger cellular signals similar to those generated due to low glucose concentrations, resulting in a starvation response rather than a fermentation response [29,30]. The results of the current study support the notion of xylose paradox and associated cellular signaling pathways, involving *GCR2* as a metabolic regulator. In the presence of *GCR2*, high concentration of xylose may trigger non-fermentative response that inhibits xylose uptake and utilization, probably due to inhibition of the pentose phosphate pathway by *GCR2*. However, deletion of *gcr2* would result in a synergy of the stimulating effect of the pentose metabolism together with bypassing the glucose and hexokinase signaling cascades, ultimately resulting in avoiding the non-fermentative response. To further test this hypothesis, genetic modification and application of biosensor strains for *GCR2* will be required.

To our knowledge, this is the first metabolic study on the roles of *GCR2* in the pentose phosphate pathway in the presence of an alternative carbon source. We showed that SR7-*gcr2Δ* increased the metabolic flow toward the pentose phosphate pathway and cellular resistance to oxidative stress. Similarly, Lamas-Maceiras et al. reported that the deletion of the *Kluyveromyces lactis GCR1* increased the pentose phosphate pathway by the activation of glucose-6-phosphate dehydrogenase (encoded by *ZWF1*), the first enzyme in the oxidative phase of the pentose phosphate pathway [31]. They found that there would be a direct regulation of the Kl*ZWF1* gene expression by *Kl*Gcr1 where the Kl*ZWF1* promoter contains a consensus sequence for Gcr1 binding. In addition, the *Δ*Kl*gcr1* strain had increased resistance to oxidative stress and glutathione species GSSG/GSH ratio, supported by other reports highlighting the importance of the pentose phosphate pathway for cellular antioxidant activity based on heterologous expressions of functional *ZWF1* from various hosts [32]. These findings support the roles of *GCR2* identified in this study, assuming that the two glycolytic transcriptional activators, Gcr1 and Gcr2, co-activate the transcription of genes involved in central carbon metabolism.

The field of mass spectrometry-based metabolome analysis has developed remarkably over the last two decades [33]. Datasets generated using this method have contributed to a more comprehensive understanding of cell processes and to an application of metabolic engineering-driven optimization of cell factories [34]. Our results also provide extensive knowledge on global metabolic changes induced by genetic modification of a glycolytic transcriptional activator. Based on the GC-MS instrumental platform, we could detect a broad range in the physico-chemically diverse metabolite pool, including sugars, organic acids, amino acids, fatty acids, and polyamines. The advantages of GC are high separation efficiency and reproducible retention times via the retention index using retention time markers [35]. However, the GC-based methodology has limitations on unsuitability for non-volatile and thermally unstable metabolites and on differentiation of sugar isomers [36]. Therefore, future studies, involving a combination of LC-MS and GC-MS-based methodologies, are required to identify metabolites more precisely.

Taken together, our study investigated the metabolic responses to xylose fermentation in *S. cerevisiae*. Based on metabolomic changes induced in the SR7-*gcr2Δ* mutant, we conclude that upregulation of the pentose phosphate pathway is the primary metabolic change observed in the SR7-*gcr2Δ* mutant when an alternative carbon source is present. These changes ultimately resulted in significantly improved xylose metabolism in the yeast strain expressing a heterologous xylose assimilation pathway. This study provides insights into how the native regulatory system *GCR2* coordinates the expression of genes in central carbon metabolism and associated metabolic pathways.

## Figures and Tables

**Table 1:** Strains used in this study.

Strains	Relevant Genotype or Descriptions	References
DX123	D452-2 *XYL1 XYL2 XYL3*	[12]
SR6	DX123 *XYL1*	[10]
SR7	SR6 *XYL2 XYL3*	[10]
BY4742 *gcr2Δ*	Yeast Knockout Collection	Thermo Fisher Scientific
SR7-*gcr2Δ*	SR7 *gcr2Δ*::KanMX4	This study

## Supplementary Materials

The following are available online at https://www.mdpi.com/2076-2607/8/10/1499/s1, Supplementary Table S1: Metabolites list, Supplementary Figure S1: Hierarchical clustering of *S. cerevisiae* metabolites.
