# Supplementary material for: Metabolic Changes Induced by Deletion of Transcriptional Regulator GCR2 in Xylose-Fermenting Saccharomyces cerevisiae
Source: Microorganisms. 2020 Sep 29;8(10):1499. doi: 10.3390/microorganisms8101499 (PMC7599485; doi:10.3390/microorganisms8101499)

[Supplementary Materials]

Metabolic Changes Induced by Deletion of Transcriptional Regulator GCR2 in Xylose-Fermenting *Saccharomyces cerevisiae*

Minhye Shin[1] and Soo Rin Kim[2,*]

[1]Department of Agricultural Biotechnology, Research Institute of Agriculture and Life Science, Seoul National University, Seoul, 08826, Republic of Korea
[2] School of Food Science and Biotechnology, Kyungpook National University, Daegu, Korea
* Correspondence soorinkim@knu.ac.kr; Tel.: +82-53-950-7769

Received: 27 August 2020; Accepted: 25 September 2020; Published: date

Supplementary Materials:

- Supplementary Table S1: Metabolites list
- Supplementary Figure S1: Hierarchical clustering of *S. cerevisiae* metabolites

Supplementary Table 1: Metabolites list

No.	Metabolite	Pubchem ID	KEGG ID
1	1-monopalmitin	14900	-
2	2-(4-hydroxyphenyl)ethanol	10393	C06044
3	2-hydroxypyridine	8871	C02502
4	2-hydroxyvaleric acid	98009	C16649
5	2-ketoadipic acid	71	C00846
6	3,6-anhydro-D-galactose	16069996	C06474
7	3-hydroxypyridine	7971	2502
8	5'-deoxy-5'-methylthioadenosine	439176	C00170
9	adenosine	60961	C00212
10	adenosine-5-monophosphate	15938965	C00020
11	adipic acid	196	C06104
12	alanine	5950	C00041
13	alpha-ketoglutarate	51	C00026
14	aminomalonate	100714	C00872
15	arabitol	94154	C00532
16	arachidic acid	10467	C06425
17	asparagine	6267	C00152
18	aspartate	5960	C00049
19	benzoic acid	243	C00180
20	beta-alanine	239	C00099
21	butane-2,3-diol nist	439888	C03044
22	capric acid	2969	C01571
23	cellobiose	10712	C00185
24	cholic acid	221493	C00695
25	citramalate	1081	C00815
26	citrate	311	C00158
27	cyano-L-alanine	13538	C02512
28	cytidine-5'-monophosphate	6131	C00055
29	erythrose-4-p	122357	C00279
30	ethanolamine	700	C00189
31	fructose	2723872	C01496
32	fructose-6-phosphate	69507	C00085
33	fumarate	5460307	C00122
34	galactinol	11727586	C01235
35	galactonic acid	128869	C00880

36	galactose	6036	C00124
37	glucose	5793	C00031
38	glucose-6-phosphate	21604864	C00092
39	glutamate	33032	C00025
40	glyceric acid	752	C00258
41	glycerol	753	C00116
42	glycerol-3-phosphate	754	C03189
43	glycine	750	C00037
44	glycolate	757	C00160
45	guanine	135398634	C00242
46	heptadecanoic acid	10465	-
47	hypoxanthine	135398638	C00262
48	inosine	135398641	C00294
49	isoleucine	6306	C00407
50	lactate	612	C00186
51	lauric acid	3893	C02679
52	citrulline	9750	C00327
53	leucine	6106	C00123
54	levoglucosan	2724705	C06478
55	L-homoserine	12647	C00263
56	lignoceric acid	11197	C08320
57	linoleic acid	5280450	C01595
58	lysine	962	C00047
59	lyxose	439240	C00476
60	malate	160434	C00149
61	mannitol	6251	C00392
62	mannose	18950	C00159
63	melibiose	440658	C05402
64	methionine	876	C00073
65	methionine sulfoxide	847	C02989
66	methylamine nist	604044	C00218
67	myo-inositol	892	C00137
68	myristic acid	11005	C06424
69	nicotinamide	936	C17729
70	N-methylalanine	5288725	C08263
71	O-acetylserine	99478	C00979
72	octadecanol	8221	-
73	oleic acid	445639	C00712

74	ornithine	6262	C00077
75	oxalate	71081	C00209
76	oxoproline	7405	C01877
77	palatinitol	11614543	-
78	palmitic acid	985	C00249
79	pelargonic acid	8158	C01601
80	pentadecanoic acid	13849	C16537
81	phenylacetic acid	999	C07086
82	phenylalanine	6140	C00079
83	phosphate	1061	C00009
84	phosphogluconate	91493	C00345
85	proline	145742	C00148
86	putrescine	1045	C00134
87	pyrrole-2-carboxylic acid	12473	C10825
88	pyruvate	107735	C00022
89	ribulose-5-phosphate	439184	C00199
90	saccharopine	160556	C00449
91	salicylaldehyde	6998	C06202
92	sedoheptulose	5459879	C02076
93	sedoheptulose-7-p	165007	C05382
94	serine	5951	C00065
95	spermidine	1102	C00315
96	stearic acid	5281	C01530
97	succinic acid	1110	C00042
98	tagatose	439312	C00795
99	terephtalic acid	7489	C06337
100	threitol	169019	C16884
101	threonine	6288	C00188
102	threose	439665	C06463
103	thymine	1135	C00178
104	tryptophan	6305	C00078
105	tyrosine	6057	C00082
106	uracil	1174	C00106
107	urate	1175	C00366
108	valine	6287	C00183
109	xanthine	1188	C00385
110	xylulose-5-p	5459820	C00231

Supplementary Figure S1: Hierarchical clustering of *S. cerevisiae* metabolites

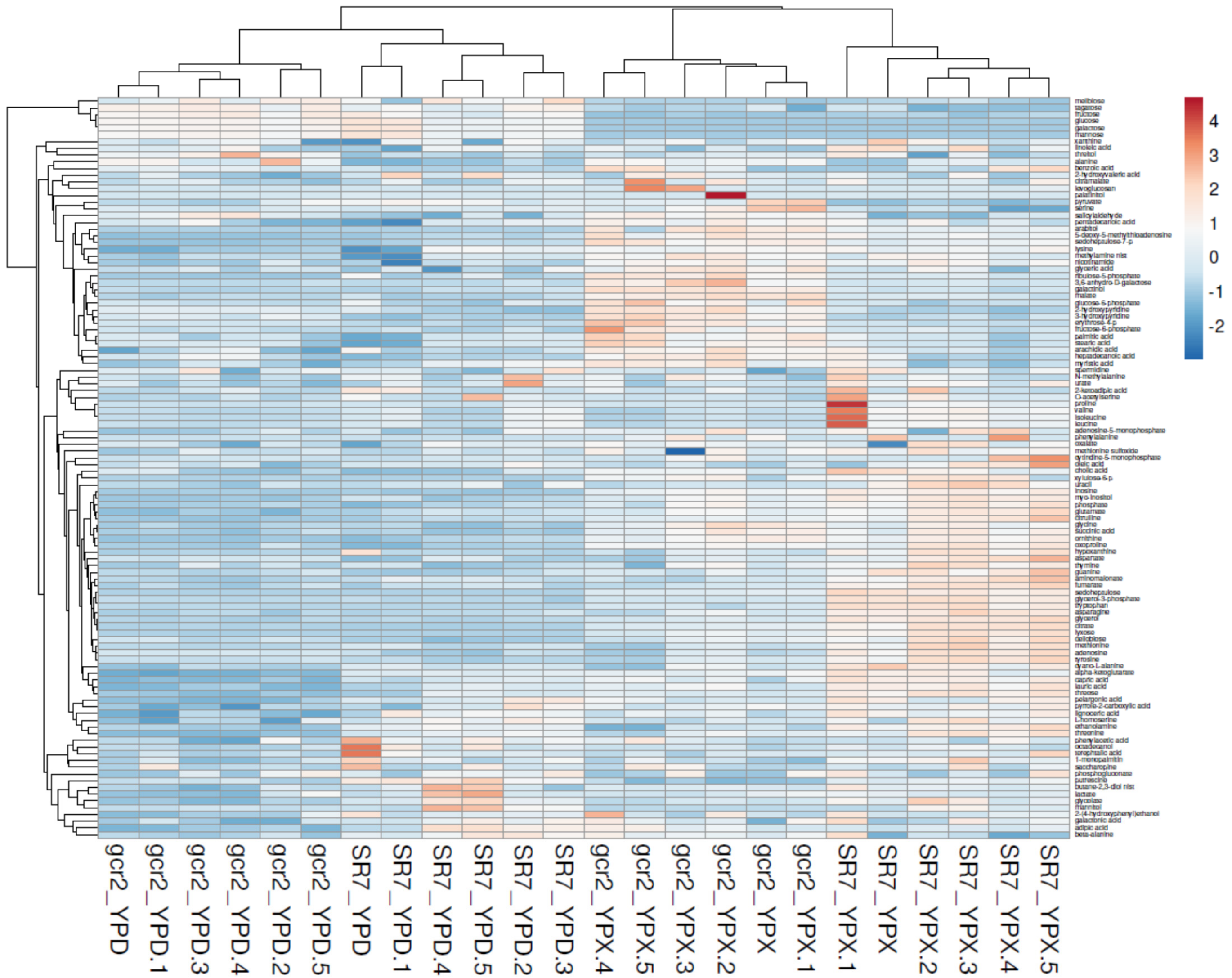

Supplement: Supplementary file 1 [file microorganisms-08-01499-s001.pdf]
